# The association between alcohol use, alcohol use disorders and tuberculosis (TB). A systematic review

**DOI:** 10.1186/1471-2458-9-450

**Published:** 2009-12-05

**Authors:** Jürgen Rehm, Andriy V Samokhvalov, Manuela G Neuman, Robin Room, Charles Parry, Knut Lönnroth, Jayadeep Patra, Vladimir Poznyak, Svetlana Popova

**Affiliations:** 1 Public Health and Regulatory Policies, Centre for Addiction and Mental Health, Toronto, Ontario, Canada; 2 Dalla Lana School of Public Health, University of Toronto, Canada; 3 Head WHO Collaboration Centre for Substance Abuse, Zurich, Switzerland; 4 Epidemiological Research Unit, Klinische Psychologie & Psychotherapie, Technische Universität Dresden, Germany; 5 In Vitro Drug Safety and Biotechnology, Department of Pharmacology, Institute of Drug Research, University of Toronto, Toronto, Canada; 6 Centre for International Health, University of Toronto, Toronto, Canada; 7 School of Population Health, University of Melbourne, Melbourne, Australia; 8 AER Centre for Alcohol Policy Research, Turning Point Alcohol & Drug Centre, Fitzroy, Victoria, Australia; 9 Alcohol & Drug Abuse Research Unit, Medical Research Council, Cape Town, South Africa; 10 Department of Psychiatry, Stellenbosch University, Cape Town, South Africa; 11 Stop TB Department, World Health Organization, Geneva, Switzerland; 12 Management of Substance Abuse, Department of Mental Health and Substance Abuse, World Health Organization, Geneva, Switzerland; 13 Factor-Inwentash Faculty of Social Work, University of Toronto, Canada

## Abstract

**Background:**

In 2004, tuberculosis (TB) was responsible for 2.5% of global mortality (among men 3.1%; among women 1.8%) and 2.2% of global burden of disease (men 2.7%; women 1.7%). The present work portrays accumulated evidence on the association between alcohol consumption and TB with the aim to clarify the nature of the relationship.

**Methods:**

A systematic review of existing scientific data on the association between alcohol consumption and TB, and on studies relevant for clarification of causality was undertaken.

**Results:**

There is a strong association between heavy alcohol use/alcohol use disorders (AUD) and TB. A meta-analysis on the risk of TB for these factors yielded a pooled relative risk of 2.94 (95% CI: 1.89-4.59). Numerous studies show pathogenic impact of alcohol on the immune system causing susceptibility to TB among heavy drinkers. In addition, there are potential social pathways linking AUD and TB. Heavy alcohol use strongly influences both the incidence and the outcome of the disease and was found to be linked to altered pharmacokinetics of medicines used in treatment of TB, social marginalization and drift, higher rate of re-infection, higher rate of treatment defaults and development of drug-resistant forms of TB. Based on the available data, about 10% of the TB cases globally were estimated to be attributable to alcohol.

**Conclusion:**

The epidemiological and other evidence presented indicates that heavy alcohol use/AUD constitute a risk factor for incidence and re-infection of TB. Consequences for prevention and clinical interventions are discussed.

## Background

Tuberculosis (TB) continues to be one of the major causes of death and disability. The Global Burden of Disease Study estimated that in 2004 TB was responsible for 2.5% of global mortality (among men 3.1%; women 1.8%) and 2.2% of global burden of disease (men 2.7%; women 1.7%), with more impact in developing countries [1]. While the current disease burden is enormous, and TB qualifies among the 10 most fatal and disabling disease categories, the relative impact of TB has been going down over time. For instance, at the time of its discovery by Robert Koch in 1882, *Mycobacterium (M.) tuberculosis*, the etiologic agent of TB, was responsible for almost one-seventh of all the deaths in Europe [2].

The association between alcohol use and TB has long been known, even before the aetiology of TB became known. Benjamin Rush as early as 1785 listed TB and pneumonia as infectious sequelae of sustained heavy drinking [3]. Since then, there have been numerous publications describing the associations between alcohol, alcohol use disorders (AUD) and TB (e.g.,[4,5]). However, the precise nature of the relationship has been in dispute. For instance, Rieder [6] (1999, p. 77) stated that "... epidemiologic evidence of a causal association [between alcohol abuse and TB] is inconclusive. The postulated association is confounded with environments (in industrialized countries at least) which are conducive for increased transmission and thus infection with M. tuberculosis." In other words, confounding by other factors could not be excluded.

We propose that, by now, enough evidence has accumulated to postulate a causal impact of alcohol use on both the incidence and the course of TB. In this paper, we will try to summarize this evidence, using the standard criteria for causality in epidemiology [7,8]: association and strength of association, temporality, consistency, dose-response relationship, plausibility of biological pathways, exclusion of confounding and alternative explanations, and reversibility following interventions. In our analysis we will rely heavily on the review and meta-analysis of Lönnroth and colleagues [9], but include new data on the association in different populations as well as two new meta-analyses on alcohol as a risk factor for TB clustering.

## Method

### Systematic literature review on epidemiological prevalence studies

AMSTAR, a measurement tool to assess the methodological quality of systematic reviews [10], and the overview by Egger and colleagues [11] were used as a guide to conduct this systematic literature review.

A systematic literature search on individual epidemiological studies reporting a prevalence of a) heavy alcohol use/AUD among TB patients and b) TB among patients with AUD was performed in multiple electronic bibliographic databases including: Ovid MEDLINE, PubMed, EMBASE, Web of Science (including Science Citation Index, Social Sciences Citation Index, Arts and Humanities Citation Index), PsycINFO, CABS (BIDS), WHOLIST, SIGLE, ETOH, Google Scholar, and the Cochrane Database of Systematic Reviews. The available published and unpublished literature was searched up to September 2008 inclusive. The search was conducted using multiple combinations of the following key words: tuberculosis, alcohol, abuse, misuse, dependence, alcohol use disorders, prevalence and incidence. In addition, a manual search of the bibliographic pages of selected articles and reviews as well as the content pages of major epidemiological journals [including The International Journal of Tuberculosis and Lung Disease (IJTLD)] were conducted. The search was not limited geographically and to English language publications.

Studies were excluded from the analysis for any of the following reasons: article did not have sufficient data on prevalence of heavy alcohol use/AUD among TB patients or of TB among patients with AUD; a meta-analysis or systematic review; duplicate publication of the same study; and articles available in abstract form only.

### Data extraction

The titles and abstracts, where available, were independently reviewed by two researchers to identify potentially relevant papers. The papers were obtained and independently read in full by two researchers. Differences were resolved by discussion and a third party, if necessary. Reasons for exclusion were identified. The data were extracted based on the inclusion and exclusion criteria defined above.

Training of coders to achieve sufficient (> 0.80) interrater reliability was conducted. Interrater reliability gives a score of how much homogeneity, or consensus, there is in the ratings given by different raters. In order to calculate interrater reliability, Fleiss' kappa statistics using the attribute agreement analytic method was used [12].

Using a standardized Excel spreadsheet, each study was coded for the following variables: reference, setting [country/study years], study sample, type of TB, percent of patients with: a) heavy alcohol use/AUD among TB patients and b) TB in patients with an AUD diagnosis, and definition of heavy alcohol use/AUD. A second member cross-checked the table entries for accuracy, against the original article.

The quality of the data from individual articles was assessed based on the following domains as recommended by U.S. Department of Health and Human Services [13]: comparability of subjects, exposure, outcome measurement, and funding or sponsorship.

### The literature search for different causal criteria and pathways

In the literature search for different causal criteria and pathways, priority was given to systematic reviews and meta-analyses. Special search strategies [14] were used. The same data bases as for the other research were used, with the key words alcohol, TB, plus one of the following: longitudinal, consistency, dose-response relationship, biological pathways, confounding, and reversibility. Only the most comprehensive reviews were included for the discussion of different causal criteria.

### Estimation of a) standardized mortality rates in Russia and the Ukraine and b) alcohol-attributable fractions (AAFs) for TB deaths

Comparison of the standardized mortality rates in Russia and the Ukraine was based on the WHO mortality data bank (European standard population).

AAFs for TB deaths in 2002 in selected countries were calculated based on relative risk (RR) information from the most recent meta-analysis [9]. AAFs were calculated using the formulas of Walter [15,16], using a disaggregated approach with 5 age groups (15-29; 30-44; 45-59; 60-69; 70+ years). For exposure, we used the estimates of heavy drinking from the 2002 Comparative Risk Analyses for WHO [17]. This model is restricted to adults and only captures the effect of alcohol on oneself, not potential effects on others in spreading the disease.

Even though these studies [14] controlled for some confounding, residual confounding of social and other risk factors for TB such as smoking or drug taking cannot be ruled out [18]. Unfortunately, there is no sufficient basis for the quantification of confounding. To be conservative, we use the standard method for potential confounding of tobacco risks (i.e. halving the effect) [19].

### Ethics committee approval

The underlying work is based on systematic reviews of published data, and thus did not require ethical review.

## Results

### Systematic literature review on epidemiological prevalence studies to establish association between heavy alcohol use/AUD and TB

**Figure 1:**
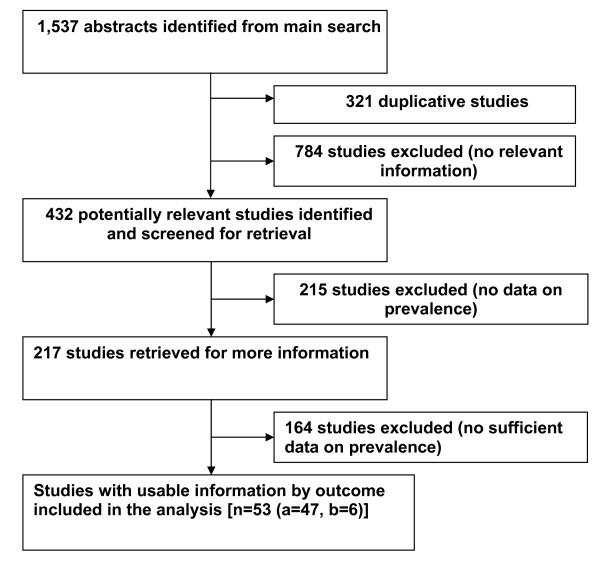
**Flow diagram describing selection of studies for a systematic review on prevalence of a) heavy alcohol use/AUD among TB patients and b) TB among patients with AUD**.

The electronic searches yielded a total of 1,537 publications regarding epidemiological prevalence studies (Figure 1).

After 321 duplicate articles were removed, 1,216 were screened using titles and abstracts. 432 full-text articles were retrieved for further consideration. Cross-checking of references and hand-searches of the IJTLD did not yield any additional studies to be included. A total of 53 studies met inclusion criteria: 47 studies reported a prevalence of heavy alcohol use/AUD among TB patients and 6 studies reported a prevalence of TB among patients with AUD. Studies were in English (50), Russian (2) and Romanian (1) languages. A list of excluded studies is available from the authors. The studies were found for the following countries: USA (29 studies), Russia (5), Canada (4), Denmark (3), India (3), Brazil (2); and one study in each of Australia, Belarus, Finland, Germany, Kazakhstan, Romania, Slovenia, Sweden, and the UK.

### The literature search for different causal criteria and pathways

At least one meta-analysis or systematic review was found for each of the causal criteria, except for establishing the dose-response relationship and reversibility. In the following, the results with respect to the causal criteria will be presented and discussed under the respective subheading. A general discussion will follow.

### Association and strength of association

There have been clear associations found between heavy alcohol use/AUD, variously defined, and TB. Additional file 1 provides an overview of the prevalence of heavy alcohol use/AUD in various samples of TB patients. The prevalence of heavy alcohol use/AUD in most samples of men and women is markedly higher than in the general population, or than in matched control groups. As expected, the prevalence of TB among people with AUD was also elevated (see additional file 2). In addition, TB was found to be a major cause of death among people with AUD [20].

A formal meta-analysis, conducted by one of the co-authors of the present manuscript, was based on 3 cohort and 18 case-control studies with individual-level data on alcohol exposure and TB [11]. The objective was to systematically review alcohol use as a risk factor for active TB. The pooled RR across the 11 studies that used an exposure cut-off level set at 40 g alcohol per day or above, or defined exposure as a clinical diagnosis of an AUD, was 3.50 (95% CI: 2.01-5.93). After exclusion of 3 small studies, because of suspected publication bias, the pooled RR was 2.94 (95% CI: 1.89-4.59). Several sensitivity analyses confirmed this substantial risk of TB for heavy alcohol use or AUD [9]. The pooled effect size across studies that had controlled for infection status (OR 4.21, 95% CI: 2.73-6.48), suggest that one possible causal pathway is through increased risk of progression from infection to disease. This meta-analysis did not find an association between consumption of less than 40 g alcohol per day and risk of TB.

Two recent meta-analyses have assessed risk factors for TB clustering (as a marker for recent TB transmission), and both found that alcohol abuse (not further defined) was a risk factor for clustering. The first, which was based on 36 genotyping studies in 17 countries [21], found significant effects of alcohol abuse on TB clustering for both low TB incidence (OR = 2.6; 95% CI: 2.1-3.3) and high TB incidence (OR = 1.4; 95% CI: 1.1-1.9) studies. The significant effects remained after adjustment [21]. The other meta-analysis, based on 30 studies, reported that recent transmission of TB was associated with heavy alcohol use (OR 2.27; 95% CI: 1.69-3.06) [18].

In summary, the evidence indicates a clear and strong association between heavy alcohol use/AUD and both risk of developing active TB and risk of recent TB transmission.

### Temporality

Most of the studies in [9] were case-control studies, where either newly detected or recurrent cases of active TB were compared to control populations. Exposure (either heavy use or AUD) was measured retrospectively in these studies, sometimes for the past year only, or for longer time periods. However, since AUD takes usually years to develop, the temporal sequence of AUD before TB seems to be confirmed in case-control studies with newly detected TB as the outcome variable. It is hardly conceivable that the AUD could have developed after the onset of active TB and before the subsequent interview (usually within weeks), for example as a reaction of the patient to being diagnosed with TB. Moreover, there are a few cohort studies where the temporality has been clearly established (see [22,23]).

Thus, the available evidence clearly supports a temporal sequence with heavy alcohol use/AUD preceding TB.

### Consistency

Overall, the association between heavy alcohol use/AUD and TB is quite consistent (see additional files 1 and 2; and the results of the three meta-analyses). The association has been replicated in studies in different countries, using different settings and different methodologies. Formal tests of consistency have been conducted in the meta-analysis of Lönnroth and colleagues [9] for the subset of studies which could give indicators of effect strength in the form of RRs. Marked heterogeneity was found when all measures of exposure were included, that is if dichotomous measures of any alcohol consumption at a time before incidence of TB (present/absent) were included [9]. This original heterogeneity decreased considerably after subdividing studies into low- and high-exposure levels (high-exposure defined as a measure of AUD and/or average consumption of ≥ 40 g/day). However, statistically significant heterogeneity persisted in the high-exposure category, which may be due to either the rather wide definition of exposure (including definitions based on average volume as well as on psychiatric classification), to misclassification of exposure across studies, to different types of control groups used, or to the different endpoints included (i.e., pulmonary TB vs. all TB; first incident TB vs. recurrent TB). While heterogeneity was statistically significant, it should be stressed that no study for heavy alcohol use/AUD showed a RR below 1, and all tests, including on various subsets of studies controlled for different confounders, showed pooled RRs higher than 3 [9].

In summary, there is a consistent relationship between heavy alcohol use/AUD and TB, and more formal measures of risk stemming from controlled studies were consistently positive with relatively large effect sizes.

### Dose-response relationship and impact of patterns of drinking

There are few studies that allow an explicit test of a dose-response relationship between alcohol use and TB. The work of Brown and Campbell [24] is the exception, and from the data provided in that paper, the following odds ratios can be calculated for different levels of exposure: 10-25 ml/day: 1.60 (95%CI: 0.57-4.55); 26-50 ml/day: 2.38 (95% CI: 0.89-6.44); 51-75 ml/day: 9.27 (95% CI: 2.77-32.58); 76-100 ml/day: 8.50 (95% CI: 1.93-40.54); >100 ml/day: 35.55 (95% CI: 6.41-260.70). However, these findings are based on a small sample of men, and the confidence intervals are wide. A large case-control study of Russian mortality data [25] published as these analyses found, in comparison to light drinkers, the following dose-response relationship beyond an apparent threshold: <1 half-litre bottle of vodka or equivalent per day relative risk 1.01 for men (95% CI 0.83-1.22), 0.93 for women (95% CI 0.64-1.35); one to less than 3 bottles 1.97 for men (95% CI 1.64-2.36), 4.06 for women (95% CI 2.97-5.56); 3 bottles and above 4.14 for men (95% CI 3.44-4.98), 5.32 for women (95% CI 3.70-7.65).

Most other studies use dichotomous exposure with some variant of alcohol "abuse", either operationalized as average heavy drinking or by using AUD as the defining criterion. Assuming an exponential dose-response relationship like that found in the studies cited above is true, one would of course expect that any dichotomous variable associated with heavy drinking would be significant. AUD are strongly associated with frequency of heavy drinking occasions, and thus with overall volume of alcohol consumption [26,27]. Thus, the current evidence does not contradict an exponential dose-response relationship between average volume of alcohol use and TB. Clarifying the exact relationship between alcohol exposure and TB should be one of the priorities of future research, including a test for gender-specific relationships. But also, independent of a diagnosis of AUD, patterns of drinking have been identified as a risk factor for a number of disease categories over and above volume of consumption [28], and this line of investigation should be extended to TB as an outcome as well.

In summary, there is some evidence for a dose-response relationship between level of alcohol use and risk for TB.

### Pathways I: Alcohol and susceptibility to tuberculosis

About one-third of the people in the world are infected with *M. tuberculosis *[29]. Ninety percent of individuals in the general population who become infected with *M. tuberculosis *will never develop clinical disease [30]. That means that the majority of people infected with *M. tuberculosis*, those who have an adequate immune system, are able to fight off the infection and do not develop the disease. Only about 10% of those infected develop active TB, where the immune system is not able to fight off the infection [31].

Research to date has shown that heavy alcohol use/AUD is a risk factor for an impaired immune system, and increases a person's susceptibility to active TB infection as well as to the reactivation of latent disease [5,31-34]. Alcohol-consuming hosts are considered "immuno-compromised" because the incidence and severity of infectious diseases among them are greater than for abstainers [5,33-36]. Individuals with alcohol dependence are particularly susceptible to lung infections such as TB and pneumonia [5,32-34].

Greater than 90% of inhaled *M. tuberculosis *bacteria are normally destroyed by alveolar macrophages [37]. If alveolar macrophages are not able to kill *M. tuberculosis*, the bacteria multiply within macrophages, and tubercles form in the lungs. *In vitro *studies have shown that intracellular survival and growth of *M. tuberculosis *within human macrophages is enhanced by exposure to alcohol [38-40]. It has also been shown that alcohol use exacerbates murine pulmonary TB, which is associated with alterations in the region-specific CD4+- and CD8+-lymphocyte responses and defective lung granuloma formation [41]. *In vivo *and *in vitro *studies have demonstrated that alcohol significantly hinders antimycobacterial defenses by suppressing mobilization, adherence, phagocytosis, and superoxide production of alveolar macrophages [42-45]. Chronic ethanol ingestion in rats significantly decreases membrane expression of the GM-CSF receptor in alveolar macrophages and, in parallel, decreases cellular expression and nuclear binding of the transcription factor that activates GM-CSF-dependent macrophage functions [46].

Acute and chronic alcohol exposure may decrease the activation of antigen-specific T-cells by inhibiting the macrophage's capacity to present mycobacterial antigen to lymphocytes [47]. In addition, alcohol has been shown to reduce macrophage response to immune system modifiers (e.g., cytokines, including interleukin-6 (IL-6), IL-1β, TNF-α, and IL-8) and to prevent the protective effect exerted by the cytokines [40,48]. Moreover, acute and chronic alcohol exposure may suppress the capacity of monocytes to produce cytokines, which directly inhibit bacterial growth and play a critical role in cellular communication, activation, proliferation, and migration, as well as regulating inflammation and healing mechanisms [48,49]. Finally, alcohol may adversely affect antigen-specific T-cell activation so that the Th2 population (humoral immunity) dominates the Th1 population (cell-mediated immunity, responsible for overcoming TB infection). This shift disturbs a balance between the two basic types of immune system, compromising the immune defense and increasing susceptibility to TB as a result of alcohol exposure [42-45,50].

The contribution of alcohol to TB through its effects on the immune system has been difficult to isolate from other adverse factors, as it is still not known whether alcohol *per se *or the other sequelae of heavy drinking and alcohol dependence such as liver damage, nutritional deficiency, or hygienic factors are primarily responsible for the impaired immunity associated with alcohol dependence [5,33,51,52]. However, by whatever mechanism, alcohol exposure clearly has an impact on weakening the immune system and thus on the incidence of active TB.

In sum, there is good evidence for a biological plausibility of causal relationship between alcohol exposure and incidence of TB via weakening the immune system.

### Pathways II: Social marginalization and drift

AUD and TB have both been labeled "diseases of poverty", and both can be consequences as well as causes of social marginalization [5,53,54]. TB incidence follows a socioeconomic gradient, where TB is more incident and prevalent in poorer areas, and among poorer households [54].

The relationship between socioeconomic status and heavy alcohol use/AUD is more complex. On the country level, higher GDP has been strongly associated with higher levels of alcohol use and lower abstainer rates [55,56]. Within countries, while lower socioeconomic strata often have higher rates of abstention, rates of AUD are not necessarily lower; but stigma and health harm attached to alcohol use have been found higher in lower compared to higher strata [56-59]. Furthermore, among specific groups such as the homeless or the imprisoned population, AUD rates are much higher than in the general population [60-65].

Causal relations are not necessarily unidirectional: poverty may lead to AUD, and AUD may thus be on the causal pathway from poverty to increased TB risk. On the other hand, AUD can lead to downward social mobility, and such downward mobility can create or reinforce social conditions that increase the risk of TB.

Social marginalization and the associated conditions such as crowding, malnutrition, homelessness, and imprisonment increases the risk of TB independently of heavy AUD [9]. This means that we should not consider all of the relationship between heavy alcohol use/AUD and TB as causal. But in most studies heavy alcohol use/AUD emerges as a significant independent risk factor for TB, even if other risk factors are controlled for [9].

In sum, there is evidence of a social marginalization and drift pathway on how alcohol could lead to TB.

### Reversibility - effect of removing heavy alcohol exposure

Unfortunately, our second search on causal criteria (see above) did not yield any controlled trials on the impact of removing heavy alcohol exposure/treatment for AUD on incidence or re-infection of TB. On the aggregate level, we can learn from the natural experiment of the Gorbachev-era campaign which reduced alcohol use in the Soviet Union [66]. In the mid-eighties, the Gorbachev government, through a variety of measures including a decrease in the state controlled production of alcohol, succeeded in markedly reducing *per capita *consumption of alcohol between 1984 and 1987 from 14.2 l to 10.7 l of pure alcohol (-24.6%). These numbers take into account unrecorded consumption such as illegally produced moonshine [67]. After the campaign, alcohol use went back to old levels (1993: 14.5 l; + 35.5% from 1987). At the same time TB mortality showed similar trends for both Russia and the Ukraine (see Table 1).

**Table 1:** Comparison of standardized mortality rates in Russia and the Ukraine (adults 20-64 years of age)

Comparison 1987/84			
Men		Women	
Russia	Ukraine*	Russia	Ukraine*
- 24.9%	- 17.9%	- 19.9%	- 21.6%
**Comparison 1993/87**			

Men		Women	
Russia	Ukraine	Russia	Ukraine
+ 70.7%	+ 37.4%	+ 41.7%	+ 26.3%

* Ukraine data were only available for 1985 instead of 1984

Basis: own calculations based on WHO mortality data bank (European standard population)

Of course, the above data are only ecologic, and alternative explanations cannot be excluded. It is reasonable to believe that dramatic changes in both socioeconomic conditions and gradients, as well as the functioning of public health care systems, contributed to the upsurge in TB incidence in Russia during the 1990s [68]. However, for the reduction of overall mortality between 1984 and 1987, there are not a lot of alternative explanations. In addition, the reduction of mortality was not uniform across all causes of death. In line with the explanation of a causal impact of alcohol, causes of death 100% attributable to alcohol (e.g. alcohol poisoning) showed higher mortality rate decreases, and causes where no impact was expected (e.g., cancer, where any impact of alcohol should be long term and no effect in the first years can be expected [69]) showed no change at all. In addition, other countries in Eastern Europe without a parallel reform did not show decreases between 1984 and 1987 (e.g., Romania showed an increase of 38.8% in men and of 13.0% in women; own calculations - see Table 1). On the other hand, for the increases in TB mortality after 1987 several explanations are possible. The Soviet Union broke apart, the economic situation in countries like Russia, Ukraine or Romania worsened, and unemployment and other indicators of social crisis increased. Thus, TB as a disease category linked to poverty would be expected to increase. It is no surprise in this situation that TB rates in a country like Romania also increased (99.0% in men, 128.1% in women).

### Alcohol in the clinical course of tuberculosis

In addition to alcohol's role in the onset of TB, there is also strong evidence of a negative influence of heavy drinking/AUD on the clinical course of TB [70-74]. People drinking heavily or with AUD show higher relapse rates [75], a higher probability of an unfavourable clinical course, and a higher probability of experiencing the most destructive forms of TB [76-78]. Recent studies have also shown that heavy drinking is positively associated with early relapse (OR 17.7) [79]. There are several reasons that the clinical course of TB is worse in those with AUD than in others - both clinical and social.

#### Immunosuppression and altered pharmacokinetics of drugs used in treatment of TB

One of the major factors in increased risk for the onset of TB and its malignant clinical course is the alteration of immunoregulation resulting in immune deficiency, which leads to increased vulnerability to bacterial agents [80]. These changes affect the immune system as a whole (see above), but, as the elimination of ethanol also occurs by exhalation, it can have local pulmonary effects [81].

Another clinical reason for a worsened course of TB in heavy drinkers is the changes of drug pharmacokinetics. In particular, studies of the pharmacokinetics of isoniazide in the treatment of TB co-morbid with alcohol dependence have shown significant decreases in absorption of the drug and its accelerated metabolism after oral administration to heavy drinkers [82]. This was a cause of a lowered maximum concentration and shorter half-life of the drug.

The treatment of TB is also more difficult to manage in HIV-infected patients, particularly with regard to pharmacological interactions. Some antibiotics used in treatment of tuberculosis (e.g. rifampin) and protease inhibitors share the CYP450 metabolic pathways that lead to significant changes of pharmacokinetics of these drugs when they are prescribed simultaneously. TB-therapies lower the concentrations of protease inhibitors: amprenavir, lopinavir/ritonavir and atazanavir [83]. Moreover, common anti-TB medications rifampicin and rifabutin lower the blood levels of nevirapine, efavirenz and delavirdine to sub-therapeutic levels. Low trough plasma levels of these anti-retroviral drugs may lead to HIV-resistance [84]. Alcohol metabolism involves oxidative and non-oxidative pathways. In the first step of oxidation, ethanol is converted to acetaldehyde. Alcohol dehydrogenase is the major enzyme. The microsomal ethanol-oxidizing system (MEOS) involves several cytochrome P450 isoenzymes, of which cytochrome P450 2E1 (CYP2E1) is the major constituent [85]. The risk of liver disease is related to the amount of alcohol consumed, but genetic factors, female sex, obesity, chronic viral hepatitis, hepatotoxins, and nutritional impairment all accelerate the disease process.

Alcohol even in small doses taken together with therapies produces interactions leading to adverse events [85]. Also, it has been shown that both TB-therapies and antiretroviral medications may have hepatotoxic effects additive to each other [83]. That may be explained by the fact that several xenobiotics that are metabolized by CYP2E1 have a synergistic effect with ethanol in the generation of liver damage.

#### Social exclusion and re-infection

It has been shown that having active TB leads to social exclusion and deterioration in the social environment - adverse effects on financial status and employment, and consequently a worsening of living conditions. This may lead to further exposure to *M. tuberculosis *in overcrowded places, including prisons [71,86-88]. Thus, another reason for a worsened course of TB is re-infection, which is common for socially derogated and/or marginalized people, in particular with alcohol dependence [89,90]. Social exclusion and alcohol dependence together also lead to delays in seeking and starting the treatment of TB, worsening its course in that way [91].

#### Interruption and other impediments of treatment

One of the major consequences of the deterioration of living and financial conditions which often accompanies alcohol dependence is an interruption of treatment [92,93]. Its rate is significantly higher in patients living with alcohol dependence and/or in fragile living conditions [73,94-96]. A recent study shows that among patients with TB who interrupted the course of treatment, 47.7% were heavy drinkers. The odds ratio for an interruption of treatment was 3.8 for heavy drinkers compared to other patients [71]. Retrospective analysis of case histories has shown a similar higher risk in alcohol users of default specifically for multidrug resistant TB treatment with a hazard ratio of 4.26 (95% CI: 2.09-8.68) [97]. These data have been corroborated by other research projects carried out on TB patients (e.g., [98]). The most recent systematic review showed a pooled odds ratio of 3.03 (95% CI: 1.84 - 4.99) for alcohol dependence as a default predictor [99].

#### Drug-resistant forms

A complex of factors -- immunosuppression, changes in drug pharmacokinetics, social deprivation and interruptions of treatment - leads to a lack of effectiveness of the treatment in general [100]. This can have adverse effects on drug effectiveness for the population as a whole. Recent studies have shown that bacteria have rapidly developed resistance to anti-TB drugs, as a result of prolonged exposure of patients to inappropriate treatment [101].

### Estimates of alcohol-attributable burden of disease of TB

Based on the assumptions of Lönnroth and colleagues [9], the proportion of TB attributable to alcohol as a risk factor in different countries is substantial, even under conservative assumptions (right two columns of Table 2 and [102]).

**Table 2:** Alcohol-attributable fractions for TB deaths 2002

	Alcohol exposure ≥ 40 g/day*		Alcohol-attributable fraction (AAF)		AAF (conservative estimate assuming 50% additional confounding)	
	**Men**	**Women**	**Men**	**Women**	**Men**	**Women**
**Russia**	56.5%	10.2%	54.3%	17.0%	37.0%	9.2%
**Nigeria**	39.3%	12.8%	45.3%	19.5%	29.1%	10.8%
**Thailand**	35.1%	2.2%	41.2%	4.5%	25.9%	2.2%
**South Africa**	24.3%	6.1%	36.8%	13.0%	22.6%	6.9%
**Brazil**	20.1%	2.2%	28.4%	4.2%	16.6%	2.1%
**China**	16.2%	0.3%	23.7%	0.6%	13.2%	0.3%
**India**	11.4%	0.1%	19.8%	0.4%	10.9%	0.2%
**Pakistan**	0.3%	0.01%	0.6%	0.0%	0.3%	0.0%

* based on [17] (for methodology see [155,156]

Basis: own calculations based on RR information from [9]

Not surprisingly, given the level of consumption [103], the highest alcohol-attributable fractions were estimated for Russia, and also Nigeria, Thailand and South Africa, especially for men. Overall, about 10% of the TB globally was estimated to be attributable to alcohol.

## Discussion

Experts from eight countries in North and South America, Australasia, Asia, and Africa, together with representatives of the World Health Organization (WHO; Department of Mental Health and Substance Abuse and Stop TB Department) and UNAIDS (Regional Support Team for Eastern & Southern Africa), met in Cape Town, South Africa in July 2008 to examine evidence relating to the linkages between alcohol use and TB and to consider potential causal impacts of alcohol use on the incidence and course of the disease. Participants reviewed the data from published and unpublished studies referred to above and specially prepared meta-analyses and reviewed information on biological pathways. There was general consensus of sufficient evidence to conclude that there is a causal linkage between heavy drinking patterns and/or AUD and the incidence of active TB for both men and women, and that these exposure categories are also causally linked to worsening of the disease course [104]. Despite this overall conclusion, there are still limitations in the underlying data as spelled out in the respective individual sections on causal criteria.

Establishing causality is only the first step in quantitatively estimating the amount of alcohol-attributable mortality and burden of disease. The current solution of halving of effects to adjust for confounding is not satisfactory, but more studies systematically including confounders are needed first. Finally, the most important advance would be interventions to reduce the incidence of alcohol-attributable TB, as well as to improve the course. There are effective treatment interventions for heavy alcohol use and AUD, and brief interventions have been shown to be effective for reducing heavy drinking [105], and there are as well effective treatments for severe alcohol dependence [106]. Appropriate measures could be routinely offered to those screened positively for heavy alcohol use and AUD. In addition, alcohol exposure at the population level can be reduced by policy measures such as increasing taxation on alcoholic beverages and decreasing the availability of alcohol through implementing a coherent liquor outlet policy [107,108]. Such measures have also been shown to reduce AUD. Given the size of the problem, effective policy measures and treatment interventions for heavy alcohol use or AUD should be implemented at this time for reducing the double burden associated with AUD and TB. Moreover, randomized clinical trials should be started to improve and tailor preventive and treatment interventions for heavy alcohol use and AUD during the treatment of TB.

## Conclusion

The epidemiological and other evidence presented indicates that heavy alcohol use/AUD constitute a risk factor for incidence and re-infection of TB. In addition, the course of the disease is worsened by alcohol use and in people with AUD. Interventions to reduce the impact of alcohol on TB should be considered.

## Competing interests

The authors declare that they have no competing interests.

## Authors' contributions

JR conceived the study, supervised all aspects of its implementation and led the writing. AVS, SP, MN and KL conducted the underlying systematic literature reviews. RR, CP, JP and VP contributed in the methodology and quantitative analysis of the study. All authors were involved with data interpretation, critical revisions of the paper and provided approval for its publication.

## Pre-publication history

The pre-publication history for this paper can be accessed here:

http://www.biomedcentral.com/1471-2458/9/450/prepub

## Supplementary Material

Additional file 1 **Overview of the prevalence of heavy alcohol use/AUD in various samples of TB patients**. Data extracted from the available literature on the prevalence of heavy alcohol use/AUD in various samples of TB patients [62,71,90,95,98,109-150]. Click here for file

Additional file 2 **Overview of the prevalence of TB in various samples of patients with AUD**. Data extracted from the available literature on the prevalence of TB in various samples of patients with AUD [78,120,151-154]. Click here for file
