# Supplementary material for: The association between alcohol use, alcohol use disorders and tuberculosis (TB). A systematic review
Source: BMC Public Health. 2009 Dec 5;9:450. doi: 10.1186/1471-2458-9-450 (PMC2796667; doi:10.1186/1471-2458-9-450)
Supplement: Additional file 1 — Overview of the prevalence of heavy alcohol use/AUD in various samples of TB patients. Data extracted from the available literature on the prevalence of heavy alcohol use/AUD in various samples of TB patients [62,71,90,95,98,109-150]. [file 1471-2458-9-450-S1.PDF]

Additional file 1. Overview of the prevalence of heavy alcohol use/AUD in various samples of TB patients

Author(s) / Year of Publication	Setting [Countrie(s) / Study Year(s)]	Study design and study population	Type of TB	% of patients with heavy alcohol use/AUD*	Definition of heavy alcohol use/AUD*
[109] Bailey et al. 1978	Jefferson County Health Department, Birmingham, Alabama, USA, Nov 1974	101 (M 71%) TB pts	Pulmonary TB	29% alcoholics (M 86%)	Brief Michigan Alcoholism Screening Test (MAST) scores of 6 or more.
[110] Bland et al. 1985	Montebello Center, Maryland, USA, 1981-1983	Hospitalized 39 (M 77%) TB pts in a non acute hospital	Pulmonary TB	Nearly 80% alcohol abusers (gdu)	Not defined
[111] Brudney & Dobkin 1991	New York City, New York, USA, Jan 1988 - Sept 1988	224 (M 79%) consecutive TB pts admitted to a large public hospital in New York	Pulmonary TB	53% with alcoholism (gdu)	Alcoholism: Daily consumption of more than 3 beers, one bottle of wine, or 1/2 pint of hard liquor & a history of alcohol-related illnesses (upper gastrointestinal bleeding, alcohol-related seizures, pancreatitis, or cirrhosis)
[112] Bumburidi et al. 2006	Kazakhstan (National), 2000-2002	Surveillance data on persons with newly reported cases of TB (n=18,979; M 63%)	Pulmonary TB	4% (762 alcohol abusers among persons with new sputum-smear positive pulmonary TB) (gdu)	Not defined
[113] Burack & Hollister 1960	Boston City Hospital, Boston, Massachusetts, USA, 1933-1958	47 (M 55%) TB pts from Harvard Medical Unit of the Boston City Hospital	TB peritonitis	43% (20 cases: M 55%) of TB peritonitis were preceded by alcoholic cirrhosis	Not defined
[114] Buskin et al. 1994	King County TB Clinic, Seattle, Washington, USA, 1988-1990	Cases (n=151): Active TB cases, aged >17 registered at TB clinic 1988- 1990 Controls (n=545): Individuals seeking care at the clinic, but no TB diagnosed (gdu)	Active TB	26% of cases & 13% of controls were heavy drinkers (gdu)	Self reported frequency of drinking & amount consumed. Heavy drinkers defined as 3 or more drinks/day or more than 5 drinks on average on each drinking occasion (drink = 14 g pure alcohol).
[115] CDC 2007	The National TB Surveillance System for 49 states & the District of Columbia (DC), USA, 1993-2005	11, 193 (M 62%) confirmed cases of TB reported annually to CDC (1993-2005)	Pulmonary TB	14% (1,526) TB pts were alcohol abusers, 2005 (gdu)	Not defined
[116]Cheung 1965	Toronto Hospital, Weston, Ontario, Canada, 1962	275 TB pts (M 67%)	Pulmonary TB	19% (53; M 94%) alcohol abusers out of 275	Not defined
[117] Crocco et al. 1976	Kings County Hospital Center, New York City, New York, USA., 1967-1969	54 TB pts (M 96%)	Pulmonary TB	83% (45) alcohol abusers (gdu)	All pts met criteria for classification as “definite alcoholics” according to the report of the Criteria Committee, National Council on Alcoholism.
[118]Cross et al. 1980	U.S. Public Health Service Cooperative Trial of Short-Course Chemotherapy of Pulmonary TB	The first 531 eligible pts enrolled in a U.S. Public Health Service Cooperative Trial of Short-Course Chemotherapy of PT (gdu)	Pulmonary TB	58% pts classified as alcoholic based on their statements. Only 18% classified as alcoholic by Brief Michigan Alcoholism Screening Test (MAST) scores (gdu)	Data were available to classify a pt as an alcoholic in 2 ways: 1) pt’s statement that he was a moderate, heavy or excessive user of alcohol or 2) pt’s score of 6 or more on a Brief Michigan Alcoholism Screening Test (MAST)

Author(s) / Year of Publication	Setting [Countrie(s) / Study Year(s)]	Study design and study population	Type of TB	% of patients with heavy alcohol use/AUD*	Definition of heavy alcohol use/AUD*
[90] Diel et al. 2002	Hamburg, Germany, 1997-1999	398 (M 71%) TB pts	Pulmonary TB	26% (105) alcohol abusers (gdu)	Alcoholism was defined as a maladaptive pattern manifested by 3 or more criteria of the WHO ICD-10 occurring at any time in the same 12-month period.
[119]Engbaek et al. 1973	Department of Pulmonary Medicine of Bispebjerg Hospital, Copenhagen, Denmark, 1969-1971	224 TB pts (gdu)	All forms of TB requiring treatment	34% (77) alcoholics (gdu)	Not defined
[120]Feingold 1976	Grady Memorial Hospital, Atlanta, Georgia, USA, 1972	All new cases of active TB reported to the Fulton County Health Department in 1972 (gdu)	Active TB	54% (81 of 205 TB pts in the outpatient clinic were alcoholics, compared with 5% (37 of 760) in a random sample of the clinic's population. TB pts diagnosed in the emergency room of the hospital had a 69% (34 of 49) rate of alcoholism, compared with an 18% in a general patient sample) (gdu)	A pt was considered to be an alcoholic if his record met one of the following criteria: 1) A description by a physician, or social worker of chronic, high intake of alcohol with behaviour associated with alcohol abuse; 2) Repeated hospital emergency & outpatient clinic visits during which drunkenness was observed; 3) Report of alcohol use coupled with the existence of organic, alcohol-associated disease (pancreatitis, cirrhosis, gastritis); 4) Diagnosis of alcoholism on a discharge summary from the Hospital.
[121]Fleming et al. 2006	St Petersburg & Ivanovo, Russia 2002-2003	A prevalence study of alcohol use & HIV risk behavior was conducted in a sample of 200 M 72%) adults: admitted to TB hospitals in St. Petersburg & Ivanovo, Russia	Active TB	62% (current alcohol abuse/dependence) (gdu)	DSM-IV criteria for current alcohol abuse or dependence
[98] Gelmanova et al. 2007	Tomsk, Siberia, Russia, 2001	Retrospective cohort study of consecutively enrolled, newly detected, smear &/or culture-positive adult TB pts initiating therapy in a DOTS programme (237 pts: M 62%)	Active TB	24% on treatment initiation noted as alcohol abusers (gdu)	Diagnosis of chronic alcoholism was confirmed by a narcologist.
[122] Goldstein et al. 1982	West Park Hospital, Toronto, Ontario, Canada, Jan 1977 & March 1980	389 (M 67%) TB pts	Pulmonary TB	28% (109) chronic alcoholism at the time of admission (gdu)	Not defined
[62]Haddad et al. 2005	Analysis included all TB cases reported from the 50 USA states & the District of Columbia from 1994 to 2003, based on reports submitted to the CDC as of March 30, 2004.	178,517 (M 87%) TB cases (11,369 – homeless; 167,148 nonhomeless)	Pulmonary TB	14% reported excessive alcohol use from the total sample of TB pts (178,517). The prevalence of excessive alcohol use was 4.6 times greater among homeless (54%) than among non-homeless (12%) persons with TB (95% CI, 4.5-4.7) (gdu)	Self-reported excessive alcohol use
[123] Hemila et al. 1999	South-western Finland, 1985-1993	26,975 M smokers participating in RCT on the effect of nutritional support with a-tocopherol+P- carotene for cancer prevention	Pulmonary TB	20% alcohol abusers (>=30g/day)	Self reported at baseline. Alcohol abuse categorized as 30 or more grams of alcohol per day

Author(s) / Year of Publication	Setting [Countrie(s) / Study Year(s)]	Study design and study population	Type of TB	% of patients with heavy alcohol use/AUD*	Definition of heavy alcohol use/AUD*
[124] Holmdahl 1967	Goteborg, Sweden, 1961-1964	1,015 (M 63%) new pulmonary TB pts	Pulmonary TB	42% alcoholics (out of 338 M middle-aged pts)	All M new cases between ages 35-64 were checked for the list of the Temperance Board. If even one notation had been made over the years before or during the year of the 1st TB positivity, the individual was designated "alcoholic"
[125] Hyman et al. 1962	Cook County Hospital, Chicago, Illinois, USA, 1962	23 (M 52%) pts with TB peritonitis	TB peritonitis	Of the 23 pts with TB peritonitis, 5 (22%) had evidence of cirrhosis, although the specific etiology of the cirrhosis was not identified in all cases (gdu)	Not available
[71] Jakubowiak et al. 2007	Ivanovo, Orel, Vladimir, Belgorod & Pskov regions & the Republic of Chuvashia, Russia, 2003	1,805 (M 73%) cases of pulmonary TB	Pulmonary TB	24% alcohol abuse from n=339: Ivanovo n= 64 (34%), Orel n=25 (11%), Vladimir n=133 (36%), Chuvashia n=0 (0%), Belgorod n=60 (17%), Pskov n=55 (21%) (gdu)	Alcohol abuse was defined as registered alcoholism or any mention of alcohol abuse in the medical record
[126] Kim & Crittenden 2005	Cook County Prison, Cook County, Illinois, USA, 1992-1998	Cases 441 (M 79%): All inmates screened positive for active TB. Control 478: Sex matched, random sample from prison population	Active TB	52% alcohol abusers (gdu)	Alcohol abuse as recorded in prison health record
[127]Kok-Jensen 1970	Copenhagen, Denmark, 1958-1960	428 M in TB department	Pulmonary TB	21% (89) alcohol abusers	89 M pts drank according to their own information on an average 5 bottles of alcoholic beverages or more daily (55 ml pure alcohol or more a day). Among these 89 pts, 28 drank 5-9 bottles of beer a day, & 61 drank 10 bottles or more daily.
[128]Kolappan et al. 2007	South India, 2001-2003	Cases (n=429): bacteriologically positive cases aged ≥15, detected during prevalence survey. Controls (n=93,516): Those not diagnosed with TB in the prevalence survey, aged ≥15	Pulmonary TB	32% M alcoholics	By tradition & native culture, the W in the study area neither smoke nor consume alcohol. The collection of data on exposure to tobacco smoking & alcohol consumption was therefore restricted to the M population. No further definition of alcoholism
[129] Krupitsky et al. 2006	St. Petersburg, Russia, 2000-2001	160 (68%) pts treated in TB hospital	Pulmonary TB	53% alcohol abusers (gdu)	Based on ICD-10 diagnosis of alcohol dependence
[130] Lennon et al. 1970	Mattapan Chronic Disease Hospital, Mattapan, Massachusetts, USA, 1964 – 1966	530 M TB pts	Pulmonary TB	66% (352 of the 530 M TB pts had been arrested on drunkenness charges; confirmed by probation records)	The pts were asked about the frequency of police arrests on drunkenness charges
[131]Lewis & Chamberlain 1963	Brompton Hospital, London, UK,, 1956	100 M TB cases	Pulmonary TB	39% regular M drinkers	Regular drinkers: 2 or more drinks per day

Author(s) / Year of Publication	Setting [Countrie(s) / Study Year(s)]	Study design and study population	Type of TB	% of patients with heavy alcohol use/AUD*	Definition of heavy alcohol use/AUD*
[132]Lillebaek et al. 1992	TB related information was registered & analyzed using hospital case records & laboratory data from Statens Serum Institut, Copenhagen, Denmark, 1992	350 (M 55%) TB pts	Pulmonary & EPTB	15% alcohol abusers in total sample (350 pts), 22% in 47 culture-positive pulmonary TB pts (gdu)	Not defined
[133]Lyons & Saltzman 1974	Kings county-Downstate Medical Center, Brooklyn, New York USA	TB pts of the Kings county-Downstate Medical Center (gdu)	Pulmonary TB	70% chronic alcoholics (gdu)	Not defined
[134]Milne 1970	Case Register of the Tuberculosis Branch of the Health department, Victoria, Australia, 1961 – 1966	2,800 TB pts (gdu)	Pulmonary TB	21% heavy drinkers (578: M 82%) out of 2,800)	10 or more drinks/day. The standard unit of drinking was based on the Victorian glass of beer, containing 7 oz, with an alcoholic content of 5%, giving a minimum consumption of 3.5 oz (80 gm) of ethyl alcohol a day in the case of heavy drinkers.
[135]Mori et al. 1992	Indian Health Service Hospital, Pine Ridge Reservation, Shannon County, South Dakota, USA, 1983-1989	46 (M 65%) TB cases: All new, active, adult (18+ yrs). Controls (n=46). Randomly selected matched for age & residence, from health care register in Reservation, where all residents are included	Pulmonary TB (76%), EPTB (37%) & two other sites (9%)	54% alcohol abusers (gdu)	Alcohol abuse was defined by chart documentation (notation of alcohol abuse or alcoholism on problem list or discharge summary), or by the presence of any alcohol-related admissions within 10 years, or any alcohol related outpatient visits within 5 years before developing TB
[136]O'Brien et al. 1959	Dr. U.E. Zambarano Memorial Hospital, Wallum Lake, Rhode Island, USA, 1954 - 1955	200 (M 82%) TB pts	185 pts pulmonary TB; 15 pts EPTB	50% alcohol abusers (M 94%)	2 drinks per day (29 pts); 3+ drinks per day (70 pts)
[95] Paixao & Gontijo 2007	Belo Horizonte, National System for Notifiable Diseases Southeastern Brazil, 2001-2002	50 (M 64%) TB pts	Pulmonary & EPTB (19%)	14% alcohol abusers (7out of 50) (gdu)	High alcohol use (weekly consumption of ethanol greater than 420 g)

Author(s) / Year of Publication	Setting [Countrie(s) / Study Year(s)]	Study design and study population	Type of TB	% of patients with heavy alcohol use/AUD*	Definition of heavy alcohol use/AUD*
[137] Pincock 1964	Central TB Clinic, Winnipeg, Manitoba, Canada, 1961-1963	306 (M 66%) adult TB pts	Active TB	10% alcoholics (M 86%). Problem drinkers: M 6%; W 1%; alcoholics: M 16%; W 4%<br><br>92% of these alcoholics were addicted before the TB disease was discovered.	*Problem drinker:* A person in whom the consumption of alcoholic beverages constitutes a problem in that it goes beyond the limits set by the cultural standards of his social group. The drinker indulges to such a degree as to cause concern to his family, friends & employers, & the extent of his drinking makes serious inroads upon his budget. *Alcoholic:* A term reserved for those considered to have a chronic disease or disorder of behaviour characterized by repeated excessive drinking to the extent that it interferes with health, interpersonal relations or economic functioning. Included in this group are those problem drinkers with physical complications such as hepatic cirrhosis, delirium tremens, Korsakoff's psychosis, & polyneuritis, & those with psychological &/or physical dependency commonly referred to as "alcoholic addiction".
[138]Rhodes et al. 1969	A National Survey of TB Hospitals, USA	The questionnaires were mailed to 367 hospitals having more than 20 beds for TB pts. 287 (78%) were returned & 258 (70%) were usable	Pulmonary TB	26% of hospitals estimated that 10% of their pts were alcoholics, 25% of the hospitals estimated that 10% to 19% of their pts were alcoholics, 15% reported 20% to 29% alcoholics, 37% reported a 30% to 59% & 7% reported estimates of 60% or more as the rate of alcoholism among their pts. (gdu)	Respondents were asked to estimate the percentage of hospitalized pts they considered to be alcoholics
[139]Rosenman & Hall 1996	New Jersey, USA, 1985-1987	Cases (n=148): All active M, HIV negative, cases over age of 35, born in USA, notified 1985-87 Controls (n=290): From Medicaid finance administration files, matched for age & race	Pulmonary TB	35% heavy drinkers among cases; 14% among controls.<br>Increased risk of TB was highest in heavy drinkers (OR=3.33, 95% CI 1.99-5.59)	Self reported. "Heavy drinking" was defined as >22 alcohol standard drinks/week
[140] Sabau et al. 2003	Judetul Mures, Romania, 1994-2002	5,402 active cases hospitalized at regional TB hospital; children & adults; 2.5% of the children co-infected with HIV (gdu)	Pulmonary TB	42% alcoholics (gdu)	Definition of alcoholic was given by the physician & recorded in the medical record; 70% of the cases smokers
[141]Salles et al. 2004	Rio de Janeiro, Brazil, 1997	163 (M 61%) TB pts	Pulmonary TB	24% alcohol abusers (gdu)	Not defined
[142] Santha et al. 2002	Tiruvallur District, South India, 1999-2000	676 (M 75%) TB pts	Active TB	25% (146) alcohol abusers (gdu)	Pts who said they habitually drank alcohol were considered to be alcoholic

Author(s) / Year of Publication	Setting [Countrie(s) / Study Year(s)]	Study design and study population	Type of TB	% of patients with heavy alcohol use/AUD*	Definition of heavy alcohol use/AUD*
[143]Selassie et al. 2005	South Carolina, USA, 1970-2001	15,464 pulmonary TB pts, of which 2.9% were recurrent disease. Cases (n=437; M 73%): All recurrent pulmonary TB cases, after at least 12 months from time of treatment completion. Controls (n=442: M 70%): Random sample of people who remained free of TB >12 months after completion, matched for year of initial diagnosis	Pulmonary TB	33% alcoholics among cases with recurrence status (gdu)	Medical records reviewed. "Alcoholism" as recorded in medical record
[144]Segarra et al. 1967	Boston Sanatorium, Boston, Massachusetts, USA, 1956-1963	828 (M 66%) TB pts	Pulmonary TB	18% chronic alcoholics (gdu)	Not defined
[145]Smith & Demone 1961	Massachusetts, USA, 1958 - 1959	TB pts admitted to institutional treatment	Pulmonary TB	28% pts with alcoholism; 5.3% in general population (gdu)	Not defined
[146]Story et al. 2007	London, UK, July 2003	TB pts living in London who were or should have been on treatment on July 1st 2003 (n=1,941: M 55%), identified from London TB register & local clinic records.	Not specified	8% (156) with alcoholism (gdu)	Not defined
[147]Thurston 1957	USA	The data from 29 Veterans Administration TB hospitals & sanatoriums	Pulmonary TB	25% (The estimated median number of alcoholics in the facilities) (gdu)	Not available
[148]Thomas et al. 2005	Tiruvallur district, Tamil Nadu, India, 2000-2001	486 (M 78%) pts with TB	Pulmonary TB	33% pts with alcoholism (gdu)	Self reported during initial treatment. Exposure was "habitual drinking", which was not defined in terms of amounts or frequency
[149]Yanova 1994	Western Siberia, Russia, 1982-1990	1,553 subjects, 398 (26%) cases of TB+ alcohol dependence (AD) & 341(22%) cases of TB+alcohol abuse (AA)	Pulmonary TB	48% alcoholics (M 61%). 91% of the pts with TB+AA/AD were alcoholics before diagnosed TB	Alcohol abuse & alcohol dependence defined as "according to the Russian Classification System, which corresponds to ICD-10 & DSM-IV Criteria"
[150]Zolnir-Dovc et al. 2003	Slovenia, 2001	306 (M 61%) cases of active TB were registered at the Central Registry for TB at the University Clinic of Respiratory & Allergic Diseases, Golnik.	73%) pts had pulmonary TB, 11% EPTB , & 16% pulmonary & EPTB	28% alcohol abusers (gdu)	Not defined

*All terms related to "alcohol use/users" are kept as in the original article

Active TB - the presence of M. tuberculosis infection with a positive chest X-ray

EPTB (extra-pulmonary TB) refers to TB outside the lungs, for example, in the lymph nodes or kidneys (blood or lymphatic spread of tubercle bacilli to parts of the body outside the lung may occur)

gdu - gender distribution is unknown

M – men; W – women

Pt(s) – patient(s)
