# Supplementary material for: The association between alcohol use, alcohol use disorders and tuberculosis (TB). A systematic review
Source: BMC Public Health. 2009 Dec 5;9:450. doi: 10.1186/1471-2458-9-450 (PMC2796667; doi:10.1186/1471-2458-9-450)
Supplement: Additional file 2 — Overview of the prevalence of TB in various samples of patients with AUD. Data extracted from the available literature on the prevalence of TB in various samples of patients with AUD [78,120,151-154]. [file 1471-2458-9-450-S2.PDF]

Additional file 2. Overview of the prevalence of TB in various samples of patients with AUD

Author(s) / Year of Publication	Setting [Countrie(s) / Study Year(s)]	Study design and study population	Type of TB	% of pts with TB among AUD patients	Definition of AUD*
[120]Feingold 1976	Fulton County, Georgia, 1972	All new cases of active TB reported to the Fulton County Health Department in 1972	Active TB	49% of all new cases of TB occurred in alcoholics (81 out of 164) (gdu)	A pt was considered to be an alcoholic if his record met one of the following criteria: 1) A description by a physician, or social worker of chronic, high intake of alcohol with behaviour associated with alcohol abuse; 2) Repeated hospital emergency & outpatient clinic visits during which drunkenness was observed; 3) Report of alcohol use coupled with the existence of organic, alcohol-associated disease (pancreatitis, cirrhosis, gastritis); &/or 4) Diagnosis of alcoholism on a discharge summary from the Hospital.
[151]Friedman et al. 1987	New York City alcoholic & drug abusing welfare population, New York City, New York, USA, 1984	2,641 welfare clients. 400 alcohol-only abusers; 342 drug abusers-only; 246 alcohol/drug abusers (total 970) (gdu)	Pulmonary TB	Out of 970 alcohol & drug abusers, 400 abuse alcohol-only - 39.8% with a positive tuberculin skin test (TBST+) - status & no evidence of disease & 18.3% with confirmed TB; alcohol & drug abuse - 33.3% with TBST+ - status & no evidence of disease & 13% with confirmed TB (gdu)	Alcoholism: average daily consumption of 1.92 or more ounces (58mL) of ethyl alcohol (e.g. 1 pint of wine, 4 cans of beer, or 3 mixed drinks) for at least 1 month, or inebriation on examination
[152]Friedman et al. 1996	New York City, New York, USA, 1984-1992	Cohort of 858 (M 82%) subjects in very-high-risk group who abuse drugs, alcohol or both was followed for 8 yrs.	Active TB	TB developed in 47 (5.5%). 3.5% (12 out of n=347) of TB among alcohol-only abusers; 6.3% (14 out of n=222 subjects) among alcohol & drug abusers (gdu)	Alcoholism: average daily consumption of 2 ounces (59 mL) of ethyl alcohol (e.g. 1 pint of wine, 4 cans of beer, or 3 mixed drinks) for at least 1 month, or inebriation on examination
[153] Jones et al. 1954	Salvation Army Men's Social Service Center in Minneapolis, Minnesota, USA, 1952-1953	405 of homeless M (70% alcohol abusers)	Active TB	The rate of active new cases of TB per 1,000 was 22.2 or 55.5 times as great as the rate of TB among the general Minneapolis population 0.4 per 1,000 in 1952.	Not defined
[154]Olin & Grzybowski 1966	Don Jail, Toronto, Ontario, Canada, 1966	For the 227 chronic drunkenness offenders chest X-ray, history of TB & previous treatment in sanatorium were taken (gdu).	Previous or active pulmonary TB	Of 227 chronic alcoholics studied in jail, 20 (9%) had a previous diagnosis of TB. Significant findings compatible with TB were noted in 34 of 202 (17%) chronic alcoholics & in two of 187 (1%) other offenders. Tuberculin testing of 98 inmates revealed that 81% of the chronic alcoholics & 33% of the other offenders had positive skin tests (gdu).	Not defined

Author(s) / Year of Publication	Setting [Countrie(s) / Study Year(s)]	Study design and study population	Type of TB	% of pts with TB among AUD patients	Definition of AUD*
[78](Shevchenko 2001)	Gomel, Belarus, 1984-1985	160 (M 93%) chronic alcoholics with TB were randomly selected & compared with TB pts without alcoholism (n is unknown)	Pulmonary TB	1998-1999:<br>*Incidence* of TB among alcoholics was 114.3 as compared to the TB pts without alcoholism 50.6 per 100,000, p < 0.01.<br>*Prevalence* of TB among alcoholics was 820.1 as compared to the TB pts without alcoholism 174.6 per 100,000, p < 0.01.<br>*Reoccurrence* of TB among alcoholics was 25.5 as compared to the TB pts without alcoholism 4.4 per 100,000, p < 0.01.<br>*Mortality* due to TB among alcoholics was 50.9 as compared to the TB pts without alcoholism 5.3 per 100,000, p < 0.01 (gdu)	Not defined

*All terms related to “alcohol use/users” are kept as in the original article

gdu - gender distribution is unknown

M – men; W – women

Pt(s) – patient(s)
